# Maternal Outcomes of WIC Recipients Before and During the COVID-19 Pandemic

**DOI:** 10.3390/ijerph22091354

**Published:** 2025-08-29

**Authors:** Arlesia Mathis, Sarah G. Buxbaum, Fran Close, Sandra G. Suther, Elizabeth Mazzio, Remelda Saunders-Jones, Fayetta Justin, Karam F. A. Soliman, Selina Darling-Reed

**Affiliations:** 1 Institute of Public Health, Florida A&M University, Tallahassee, FL 32307, USA; sarah.buxbaum@famu.edu (S.G.B.); fran.close@famu.edu (F.C.); 2 Economic, Social, and Administrative Pharmacy, Florida A&M University, Tallahassee, FL 32307, USA; sandra.suther@famu.edu; 3 Pharmaceutical Sciences, Florida A&M University, Tallahassee, FL 32307, USA; elizabeth.mazzio@famu.edu (E.M.); karam.soliman@famu.edu (K.F.A.S.); selina.darling@famu.edu (S.D.-R.); 4 Family Practice, 1725 Capital Circle, NE, Suite 305, Tallahassee, FL 32301, USA; remelda.saunders@famu.edu; 5 Community Outreach, 2770 Capital Medical Blvd., Tallahassee, FL 32308, USA; fayetta.justin@hcahealthcare.com

**Keywords:** WIC programs, COVID-19 pandemic, prenatal care, gestational diabetes, gestational hypertension

## Abstract

The effects of the COVID-19 pandemic restricted the availability of healthcare and social services. This retrospective study reports WIC enrollment rates and presents descriptive data on prenatal care access and selected maternal health conditions among pregnant women in Florida before and during the COVID-19 pandemic. Using birth data linking maternal and infant characteristics from the Florida Department of Health Bureau of Vital Statistics, we examined birth records from 1 January 2019 to 31 December 2020 related to women ranging from 11 to 59 years of age who received WIC. The descriptive results show that WIC recipients had higher rates of inadequate prenatal care and adverse maternal health outcomes during the pandemic. Logistic regression results show that the odds of receiving inadequate prenatal care increased by 24% (OR = 1.24, *p* < 0.001), the odds of experiencing gestational diabetes by 9% (OR = 1.09, *p* < 0.001), and the odds of experiencing gestational hypertension by 10% (OR = 1.10, *p* < 0.001). Further research is needed to evaluate how specific WIC services influence maternal outcomes, particularly during public health emergencies.

## 1. Introduction

The COVID-19 pandemic disrupted healthcare systems and social safety nets, with significant implications for maternal and infant health. Across the United States, the Special Supplemental Nutrition Program for Women, Infants, and Children (WIC) serves as a critical lifeline for low-income pregnant and postpartum individuals, infants, and young children. This nutrition program protects at-risk populations from nutrition and health-related problems such as food insecurity [1], obesity [2], and unhealthy birth outcomes [3]. However, since March 2020, the effects of the COVID-19 pandemic have placed additional financial stress on households, creating an even greater need for nutritional support programs [4,5].

### 1.1. WIC’s Role in Nutritional and Healthcare Support

Families living in lower-income communities face increasing challenges related to insufficient access to food-related resources [6]. Among these low-income populations, pregnant women face the highest risk due to their need to stay healthy for both themselves and their developing fetus. WIC provides food vouchers, nutritional education, breastfeeding support, and referrals to healthcare services [7]. Prior studies demonstrate its efficacy in reducing rates of preterm birth, low birth weight, and infant mortality. WIC participation is associated with improved dietary intake of essential nutrients (e.g., iron, calcium) during pregnancy and enhanced access to prenatal care [8]. A research brief from the Robert Wood Johnson Foundation reported that a USD 1 investment in WIC yields an estimated USD 2.48 in healthcare savings by preventing adverse birth outcomes [5].

According to the Food and Nutrition Service, approximately 11.9 million people participate in WIC each month across the United States [9]. WIC has historically mitigated racial and socioeconomic disparities in maternal health. Black and Hispanic participants, who face higher risks of preterm birth and maternal mortality, experience significant benefits from WIC’s services. However, structural barriers, such as in-person certification requirements and limited clinic hours, have historically constrained access for vulnerable populations. In addition, coverage rates vary across the U.S. by race and ethnicity. In Florida, Hispanic/Latinx populations have a coverage rate of 62.4% of those who are eligible, non-Hispanic African-Americans have a coverage rate of 59.9%, and the non-Hispanic White population has a coverage rate of 45.5% [9]. National data from Medicaid-covered births from 2016 to 2022 revealed a stark decline in WIC receipt, decreasing from 66.6% pre-pandemic to 57.9% post-pandemic [10]. Reductions were most pronounced among American Indian/Alaska Native (13.6% decrease), Native Hawaiian/Pacific Islander (22.7%), Black (15.1%), and Hispanic (11.9%) individuals [10]. These declines occurred despite increased eligibility due to rising unemployment and food insecurity [11].

### 1.2. Inequities in WIC’s Impact

Racial inequities among low-income WIC-eligible women are a public health concern. Pre-pandemic, Black and Hispanic/Latinx women participated in WIC at higher rates than White women [10]. However, declines in WIC enrollment during the COVID-19 pandemic were disproportionately greater for Black and Hispanic groups, widening existing gaps in maternal health. The COVID-19 pandemic also resulted in reduced healthcare visits. WIC’s nutritional assessments are often conducted during prenatal appointments. Inadequate access to WIC services is linked to poor outcomes in low-income African-American populations [12]. Maternal mortality rose sharply during the pandemic, increasing from 24.9 deaths per 100,000 live births in 2020 to 33.2 in 2021 [13]. Black women were disproportionately more affected during the COVID-19 pandemic, with a mortality rate 2.6 times higher than that of White women.

Although WIC participation is associated with earlier prenatal care and reduced gestational diabetes [14], declining enrollment in WIC programs among minoritized groups created less opportunity for intervention. One of the largest predictors of poor birth outcomes for both African-American and White women is lower education levels. Women with low literacy are also more likely to have a low birth weight or preterm baby. However, individuals with higher socioeconomic status have higher incomes, better jobs, more wealth, longer lives, and fewer health problems than those who are not financially well-off [6]. Studies show that women with higher education levels are more likely to receive prenatal care and receive prenatal vitamins with folic acid. However, African-American mothers with higher education are still at greater risk of having a low-birth-weight or preterm baby [15].

### 1.3. Study Aims

The purpose of this study is to examine maternal health characteristics and prenatal care access among WIC recipients living in Florida before and during the COVID-19 pandemic. Previous research has shown that sociodemographic factors contribute to a lack of access to WIC programs and prenatal care in low-income communities and among people of low socioeconomic status. The effects of the COVID-19 pandemic in 2020 restricted the availability of healthcare and social services during that year.

## 2. Materials and Methods

### 2.1. Study Data

In this study, we obtained 426,869 birth records from the Florida Department of Health Bureau of Vital Statistics recorded between 1 January 2019 and 31 December 2020 via a data use agreement. The data included all births from women identified as White, African-American/Black, and Hispanic. The vital statistics birth certificate data linked both maternal and infant information containing over 80 variables, including the demographic characteristics of the mother, as well as detailed information on the mother’s BMI, weight gain during pregnancy, prenatal visits, and chronic and pregnancy-related diseases. The linked birth record also included information on the infant, such as birth weight, congenital anomalies, infections, or other harmful exposures. Although the birth certificate data did not contain information on income, insurance information was available and used as a proxy measure for income. During early prenatal visits, all women are screened for services such as Healthy Start and WIC. This screening information is contained on the birth record in addition to whether or not the women are receiving these services. Pregnant women receiving Medicaid were eligible for WIC; however, not all eligible women participated in the WIC program. The adequacy of prenatal care was assessed using the Kessner index [16]. According to this index, inadequate prenatal care occurs if one of the following five conditions is met: (1) no prenatal care by the 16th week of pregnancy, (2) one prenatal care visit or less by the 20th week, (3) two or less visits by the 28th week, (4) three or less visits by the 32nd week, or (5) four or less visits by the 34th week. An individual who does not meet any of these conditions is considered to have received adequate prenatal care.

### 2.2. Reference Information and Artificial Intelligence Use

While preparing this manuscript, the authors used Scispace.ai for the literature review for this study. Scispace.ai was used to supplement the articles and reference information found through a traditional literature review using PubMed.

### 2.3. Data Analysis

The authors conducted descriptive analyses to examine variables of interest, which included sociodemographic characteristics, socioeconomic factors, maternal health status, and birth outcomes before and during the COVID-19 pandemic. This study retained information on 172,267 pregnant women who received WIC services for analysis. Crosstabulation analyses were conducted among the variables of interest for the period before the COVID-19 pandemic and during the pandemic. Pre-pandemic births included those from 1 January 2019 to 29 February 2020. During the pandemic, births were included from 1 March 2020 to 31 December 2020.

In addition to descriptive analysis, the authors conducted logistic regression to examine prenatal care access and two maternal health outcomes before and during the COVID-19 pandemic. We coded the time before the COVID-19 pandemic using the calendar dates from 1 January 2019 to 29 February 2020 as zero and the time during the pandemic using the calendar dates from 1 March 2020 to 31 December 2020 as one. We used data from before and during the COVID-19 pandemic as our dependent variable. We controlled for sociodemographic variables, including race, age, education, marital status, and insurance type. We examined two nutrition-related maternal health outcomes (gestational diabetes and gestational hypertension), as well as prenatal care access (adequate/inadequate). Crosstabulation and logistic analyses were conducted using SPSS, version 29.

## 3. Results

Table 1 presents the descriptive characteristics of the study population, both before and during the COVID-19 pandemic. WIC enrollment declined from 88,962 (56.8%) before the pandemic to 67,746 (43.2%) during the pandemic.

### 3.1. Sociodemographic Characteristics

Among WIC participants, the racial distribution was primarily Hispanic/Latinx (38.8% pre-pandemic, 39.0% during), followed by African-American/Black (32.9% pre-pandemic, 32.4% during) and White women (28.3% pre-pandemic, 28.6% during). Most WIC-enrolled mothers were aged 19–29 (59.5% pre-pandemic, 58.3% during); in addition, pregnancies for those women < 18 years decreased in the WIC group (4.0% pre-pandemic, 3.9% during). Most WIC participants had a high school diploma or less, with 45.1% holding a high school diploma pre-pandemic and 44.9% during the pandemic. In contrast, less than 10% of WIC recipients held a college degree or higher. These figures highlight a significant disparity in educational attainment among those receiving WIC.

### 3.2. Health Status

Pre-pregnancy BMI data showed that a greater proportion of WIC participants were obese (26.0% pre-pandemic, 26.9% during) or severely obese (6.6% pre-pandemic, 6.9% during). WIC recipients also had a higher proportion of inadequate or excessive pregnancy weight gain. Preterm birth was more frequent among WIC participants (10.9% pre-pandemic, 10.6% during). Low and very low birth weight was slightly more common among WIC participants pre-pandemic (low: 8.0%; very low: 1.5%). WIC participants showed high rates of gestational diabetes both pre-pandemic and during the pandemic (6.7%, 7.4%). In addition, WIC participants showed high rates of gestational hypertension both pre-pandemic and during the pandemic (7.8%, 8.7%).

### 3.3. Prenatal Care Access

During the pandemic, rates of inadequate prenatal care increased in WIC participants to 5.9% from 6.1%.

### 3.4. Prenatal Care Access and Nutrition-Related Health Outcomes Before and During the Pandemic

Table 2 shows the logistic regression of sociodemographic characteristics, maternal health outcomes, and prenatal care access before versus during the COVID-19 pandemic for pregnant women receiving WIC. The logistic regression shows that Black women were 3% less likely to be enrolled during the pandemic (*p* = 0.03) compared with White women, while differences between White and Hispanic women were not significant (*p* = 0.45). Additionally, women aged 30 to 39 were 10% less likely to be enrolled during the pandemic (*p* < 0.001) compared to women aged 19 to 29. When compared by education level to women with master’s degrees or above, women with less than a high school education were 12% less likely to be enrolled during the pandemic (*p* < 0.001). When examining the results from before and during the pandemic, pregnant women who are single were 3% more likely to be enrolled during the pandemic (*p* = 0.01) compared to married women. Further, pregnant women also receiving Medicaid were 13% less likely to receive WIC during the pandemic (*p* < 0.001) compared with pregnant women with private health insurance. Finally, the results also show that during the pandemic, pregnant women were 24% more likely to receive inadequate prenatal care (*p* < 0.001) compared with women receiving adequate services, 9% more likely to have gestational diabetes (*p* < 0.001), and 10% more likely to have gestational hypertension (*p* < 0.001).

## 4. Discussion

Our study examines maternal health outcomes and prenatal care access among pregnant women receiving WIC before and during the early stages of the COVID-19 pandemic. The larger sample size allowed us to gather more precise estimates of the effects of the WIC program on various populations. Moreover, compared to previous studies, we used data from before and during the pandemic, with effort focused on controlling for socioeconomic factors that contribute to poor maternal outcomes. Although other studies have evaluated the effect of WIC, our study examines this effect on over 172,000 women giving birth in a large, diversely populated state before and during the COVID-19 pandemic. In addition, this study used birth certificate data linked from the mother to infant. Researchers found only a few other studies during their literature review that used linked birth data.

Our research showed decreases in the number of pregnant women enrolled in the WIC program during the COVID-19 pandemic. This may have been due to operational barriers such as clinic closures or reduced availability of staff. Prior to the pandemic, WIC certifications or recertifications required in-person appointments. Early in the pandemic, many WIC clinics discontinued traditional in-person services [17]. To combat the discontinuation of services, between March 2020 and February 2021, the USDA Food and Nutrition program issued waivers to provide flexibility to states to enable continued access to WIC services by allowing virtual services. However, many eligible individuals were unaware of the procedural changes [18].

Our research also shows that during the pandemic, pregnant women were more likely to receive inadequate prenatal care. In our study, we defined inadequate prenatal care as women receiving fewer than the expected number of prenatal care visits using the Kessner index [16]. A reduction in prenatal visits creates additional problems because nutritional risk assessments are often conducted during prenatal appointments [10]. In addition, our study found that during the COVID-19 pandemic pregnant women were more likely to have gestational diabetes. This result was also found in a study conducted by Garrow et al. [19] for pregnant Appalachian women in West Virginia. Some known risk factors for developing GDM include excessive weight gain and obesity. Our population showed a significant number of women post-pandemic who were obese (26.9%) or severely obese (6.9%). These numbers actually increased compared to the pre-pandemic numbers (26.0% and 6.6%, respectively). Finally, our study showed that pregnant women were also more likely to have gestational hypertension. A study by Jackson et al. [20] also found increases in gestational hypertensive disorders.

Despite the abundance of data and the strength of the analysis, there are some limitations within the analysis. Data were not available regarding when the women enrolled on the WIC program or for how long the women were enrolled. In addition, some women received assistance from other pregnancy support programs, such as Healthy Start. In addition, although most of the women who received WIC were enrolled in Medicaid, the researchers did not distinguish the differences in the Medicaid recipients. For example, some women received emergency Medicaid, which allowed them to receive support for only 60 days, while other women received traditional Medicaid, which is a long-term program in which women can be enrolled for the duration of the pregnancy. Another group of Medicaid women received Medicaid managed care (MMC). Medicaid managed care provides even more support services, such as depression screening, tobacco use screening and treatment, and screenings for sexually transmitted infections. The differences in these Medicaid programs may also have unmeasured effects on our analysis. Despite the differences in the Medicaid programs, we could still see differences in birth outcomes between the women who received WIC and the women who did not when adjusted by sociodemographic factors.

Lastly, our study covered multiple years of data. The researchers did not adjust for women who may have given birth to multiple babies or may have had more than one pregnancy between 2019 and 2020. However, we believe that these limitations do not cause large biases in the analysis of the data. Future studies should explore the lingering effects of inadequate prenatal care during the COVID-19 pandemic. The higher rates of gestational diabetes and hypertension may result in a greater likelihood of the mother developing chronic conditions, as well as developmental and health impacts on the infants.

## 5. Conclusions

This study examined maternal health outcomes among women enrolled in WIC before and during the COVID-19 pandemic. WIC participants were more likely to have low incomes, be younger, and come from minoritized racial and ethnic groups; they also had higher rates of GDM, hypertension, and adverse birth outcomes. Further research should explore utilization of specific WIC services and its effects on birth outcomes.

## Figures and Tables

**Table 1:** The characteristics of WIC recipients before and during the COVID-19 pandemic.

Characteristics	Before COVID-19	During COVID-19	Total
	Number (%)	Number (%)	Number (%)
**WIC**			
Enrollment	88,962 (56.8)	67,746 (43.2)	156,708 (100)
**Race**			
Hispanic/Latinx	33,523 (38.8)	25,662 (39.0)	59,185 (38.9)
African-American/Black	28,443 (32.9)	21,333 (32.4)	49,776 (32.7)
White	24,489 (28.3)	18,802 (28.6)	43,291 (28.4)
**Age**			
≤18	3590 (4.0)	2652 (3.9)	6242 (4.0)
19–29	52,929 (59.5)	39,519 (58.3)	92,448 (59.0)
30–39	29,846 (33.5)	23,449 (34.6)	53,295 (34.0)
≥40	2597 (2.9)	2126 (3.2)	4723 (3.0)
**Education**			
<High School	15,768 (17.9)	11,518 (17.1)	27,286 (17.6)
High School Grad/GED	39,623 (45.1)	30,220 (44.9)	69,843 (44.9)
College/Associate’s Degree	24,708 (27.8)	19,000 (28.2)	43,943 (28.2)
College Graduate	6272 (7.1)	5241 (7.8)	11,513 (7.4)
Master’s Degree or Above	1675 (1.9)	1370 (2.0)	3045 (2.0)
**Marital Status**			
Single	60,466 (68.0)	46,314 (68.4)	106,780 (68.1)
Married	28,489 (32.0)	21,429 (31.6)	49,918 (31.9)
**Insurance**			
Medicaid	70,731 (79.5)	54,249 (80.1)	124,980 (79.8)
Private Insurance	11,988 (13.5)	9566 (15.6)	21,554 (13.8)
Self-Pay	3666 (4.1)	2340 (3.5)	6006 (3.8)
Other/Unknown	2577 (2.9)	1591 (2.3)	4168 (2.7)
**BMI of Mother**			
Underweight	3090 (3.7)	2146 (3.3)	5236 (3.5)
Normal	30,449 (36.0)	22,960 (35.3)	53,409 (35.7)
Overweight	23,464 (27.8)	17,960 (27.6)	41,424 (27.7)
Obese	21,943 (26.0)	17,508 (26.9)	39,451 (26.4)
Severe Obesity	5557 (6.6)	4517 (6.9)	10,074 (6.7)
**Pregnancy Weight Gain**			
Lost Weight	2150 (2.5)	1651 (2.5)	3801 (2.5)
Low Gain	26,248 (30.7)	19,648 (29.7)	45,896 (30.2)
Normal Gain	50,132 (58.5)	38,989 (58.9)	89,121 (58.7)
Excess Gain	7098 (8.3)	5901 (8.9)	12,999 (8.6)
**Gestation**			
Preterm	9669 (10.9)	7176 (10.6)	16,845 (10.8)
Full Term	79,275 (89.1)	60,556 (89.4)	139,831 (89.2)
**Birth Weight of Infant**			
Very Low	1294 (1.5)	887 (1.3)	2181 (1.4)
Low	7104 (8.0)	5352 (7.9)	12,456 (7.9)
Normal	75,252 (84.6)	57,402 (84.7)	132,654 (84.7)
High	5303 (6.0)	4100 (6.1)	9403 (6.0)
**Inadequate Prenatal Care**			
Yes	5249 (5.9)	4147 (6.1)	9396 (6.0)
No	83,713 (94.1)	63,599 (93.9)	147,312 (94.0)
**Gestational Diabetes (GDM)**			
Yes	5914 (6.7)	5009 (7.4)	10,923 (7.0)
No	82,928 (93.3)	62,659 (92.6)	145,587 (93.2)
**Gestational Hypertension**			
Yes	6966 (7.8)	5869 (8.7)	12,835 (8.2)
No	81,876 (92.1)	61,799 (91.3)	143,675 (91.8)

**Table 2:** Logistic regression of WIC recipients before and during the COVID-19 pandemic.

Characteristics	OR	95% CI	*p*-Value
**Race**			
Black	0.97	0.95–0.99	0.03
Hispanic	1.01	0.98–1.03	0.45
White	Reference	-	-
**Age**			
≤18	0.90	0.83–0.97	0.01
19–29	Reference		
30–39	0.90	0.85–0.95	<0.001
≥40	0.94	0.89–0.99	0.04
**Education**			
<High School	0.88	0.82–0.95	<0.001
High School Grad/GED	0.92	0.86–0.99	0.03
College/Associate’s Degree	0.91	0.85–0.98	0.01
College Graduate	1.02	0.94–1.10	0.72
Master’s Degree or Above	Reference	-	-
**Marital Status**			
Single	1.03	1.01–1.05	0.01
Married	Reference	-	-
**Insurance**			
Medicaid	0.87	0.85–0.90	<0.001
Private Insurance	Reference		
Self-Pay	0.75	0.71–0.79	<0.001
Other/Unknown	0.74	0.69–0.79	<0.001
**Inadequate Prenatal Care**			
Yes	1.24	1.20–1.29	<0.001
No	Reference	-	-
**Gestational Diabetes**			
Yes	1.09	1.04–1.13	<0.001
No	Reference	-	-
**Gestational Hypertension**			
Yes	1.10	1.06–1.14	<0.001
No	Reference	-	-

## Data Availability

The data that supported the findings of this study were provided by the Florida Department of Health Bureau of Vital Statistics, but restrictions applied to the availability of these data, which were used via a data use agreement (DUA #20211016) for this study, so they are not publicly available. Data are, however, available from the Florida Department of Health Bureau of Vital Statistics (https://www.floridahealth.gov/statistics-and-data/data-and-statistics/index.html).

## Abbreviations

The following abbreviations are used in this manuscript:

WIC	Women, Infants, and Children Programs or Services
COVID-19	Coronavirus Disease of 2019
MMC	Medicaid Managed Care
GDM	Gestational Diabetes Mellitus
USDA	United States Department of Agriculture
